# Investigation of Histamine, Physicochemical Quality, and Potential Health Risks in Various Canned Fish Products

**DOI:** 10.3390/foods14132314

**Published:** 2025-06-30

**Authors:** Sena Tunç, Burak Demirhan, Buket Er Demirhan

**Affiliations:** 1 İzmir City Hospital, 35540 Bayraklı, İzmir, Türkiye; senatunc97@hotmail.com; 2 Department of Pharmaceutical Basic Sciences, Faculty of Pharmacy, Gazi University, 06330 Ankara, Türkiye; bdemirhan@gazi.edu.tr

**Keywords:** canned fish, histamine, risk, quality

## Abstract

An investigation of the histamine presence in canned fish is crucial in terms of food safety and human health. The aim of this study was to investigate the levels of histamine, pH, and salt in various canned fish products consumed in Ankara, Türkiye. For this purpose, a total of 80 canned food samples (30 tuna, 30 sardine, and 20 anchovy samples) from different firms were analyzed for histamines using an enzyme-linked immunosorbent assay technique. In 33 (41.25%) out of 80 food samples, histamine was detected in concentrations ranging between 2.51 mg/kg and 20.97 mg/kg. The mean histamine levels (±SE) of the canned tuna, sardine, and anchovy samples were found to be 7.05 ± 1.07 mg/kg, 4.09 ± 0.32 mg/kg, and 4.67 ± 0.58 mg/kg, respectively. The mean pH levels (±SE) of the canned tuna, sardine, and anchovy samples were found to be 5.91 ± 0.009, 6.32 ± 0.02, and 5.99 ± 0.04, respectively. At the same time, the mean salt levels (±SE) of the canned tuna, sardine, and anchovy samples were found to be 1.18 ± 0.03%, 1.80 ± 0.09%, and 1.91 ± 0.09%, respectively. Our data reveal that the histamine levels found in all the samples were within the Turkish Food Codex values. In addition, the mean values of pH and salt found in the canned fish samples were within the Turkish Standard Institute values.

## 1. Introduction

Seafood represents an essential food source for people [1]. Fish and fish products are valuable animal protein sources for human nutrition [2]. In addition, fish contains minerals (calcium, phosphorus, magnesium, iron, zinc, selenium, fluorine, and iodine), vitamins (A, E, D, and some B group vitamins), and important fatty acids [3,4,5].

Fresh and processed fish products are highly perishable. Depending on the quality of the fish and the storage conditions, its shelf life can extend from a few days to a few weeks [6]. Therefore, various preservation methods, such as canning, freezing, salting, smoking, and drying, are used to increase access to seafood and extend its shelf life [6,7,8]. Canning is one of the most popular ways to preserve fish. In 2021, approximately 18.6 million tons of the total worldwide fisheries production (182.05 million tons) was preserved by canning. The most important types of canned fish are tuna, anchovies, bonito, sardines, and mackerel [9]. These foods are highly susceptible to biogenic amine formation due to the metabolism of microorganisms, which causes quality loss and spoilage [5]. Biogenic amines are considered contaminants of foodstuffs and cause poisoning. They are low-molecular-weight nitrogen compounds with biological activity produced by specific microorganisms (bacteria, yeast, and molds) due to the metabolism of some free amino acids, usually through enzymatic reactions of a microbial origin. These enzymatic reactions can be the decarboxylation, reductive amination, or transamination of specific precursor amino compounds [10]. The intake of low amounts of biogenic amines through food is not normally harmful to health, as they can be detoxified in the intestine. However, when the amount of biogenic amines in food is too high or when the detoxification ability in humans is blocked or impaired, they can turn into toxic metabolites that are responsible for serious health problems [11]. In addition, some biogenic amines, such as histamine and tyramine, play essential physiological roles in humans. Histamine is involved in processes such as neurotransmission, hematopoiesis, synaptic transmission, allergic responses, the regulation of cell proliferation, and others [12].

Histamine is the common and most crucial bioactive amine in different foods and beverages [13]. This biogenic amine is found in different amounts in various foods, such as fish and fish products, cheese, wine, sausages, fermented meats, and vegetables [10]. High amounts of histamine can form in fish rich in free-histidine amino acids, such as tuna, mackerel, bonito, and anchovies [10,14,15,16,17]. Histamine occurrence in fish is associated with bacterial spoilage. Therefore, histamine can be a useful indicator for estimating fish’s freshness or degree of spoilage [18]. Histamine intolerance or enteral histaminosis is a metabolic disorder characterized by a heightened sensitivity to dietary histamine resulting from a reduced histamine degradation capacity, typically associated with decreased diamine oxidase (DAO) enzyme activity [19]. On the other hand, histamine degradation is mainly carried out by the enzyme DAO or histamine N methyltransferase (HNMT). Therefore, chemical or biological histamine degradation by DAO or the addition of bacteria capable of degrading histamine can also be a preventive measure [20]. In some cases, DAO enzyme activity is not enough to affect the metabolism of even low levels of biogenic amines due to gastrointestinal diseases or secondary effects of drugs or alcohol. Some biogenic amines, such as histamine and tyramine, pose a health risk to sensitive individuals, which can be significant when other substances enhance their effects [21]. Fish species belonging to the Scombridae (mackerel, tuna, bonito) and Scomberesocidae (saury) families, also known as scombroid fish, are the species most frequently encountered in histamine (scombroid) poisoning cases due to the high free-histidine content in their muscles [22,23]. This poisoning arises from the consumption of fatty fish contaminated with bacteria that induce the production of high histamine levels. Various Gram-negative bacteria such as *Morganella morganii*, *Klebsiella pneumoniae*, and *Enterobacter* spp. have been identified as histamine producers in scombroid fish [22].

The aim of this study was to determine the histamine levels in canned tuna, sardine, and anchovy samples marketed in Ankara, Türkiye, using the enzyme-linked immunosorbent assay (ELISA) technique, as well as to analyze their pH and salt content as additional quality parameters. There is a limited number of similar studies that have evaluated the histamine levels in canned fish products in Türkiye. Therefore, this study investigated various types of canned fish, which is crucial for detecting the presence of histamine, a factor that can adversely affect food safety and quality.

## 2. Materials and Methods

### 2.1. Samples

In the present study, a total of 30 canned tuna samples (10 from each of three different firms, labeled A, B, and C), 30 canned sardine samples (15 from each of two different firms, labeled D and E), and 20 canned anchovy samples (10 from each of two different firms, labeled F and G) were analyzed. All 80 canned fish samples used in our study were produced in Türkiye. The canned food samples were obtained from supermarkets in different districts of Ankara and brought to the laboratory. The packaging of these food samples was visually examined, and samples with undamaged packaging were used for the analysis. Care was taken to ensure that the production dates and serial numbers of the supplied canned foods differed.

### 2.2. Histamine Analysis

The analysis of histamine in the canned fish samples was performed using a commercial competitive enzyme-linked immunosorbent assay (ELISA) kit (Ridascreen^®^ Histamine, Art. No. R1601, R-Biopharm AG, Darmstadt, Germany), following the manufacturer’s instructions. All reagents, including the washing buffer, controls, conjugate, substrate/chromogen, anti-histamine antibody solution, and stop solution, were supplied as ready-to-use components in the kit. The competitive ELISA was based on an antigen–antibody reaction. The wells of the microtiter strips were coated with histamine [24]. The canned fish sample was homogenized. A total of 1 g of the homogenate was weighed, and 9 mL of distilled water was added and mixed with the homogenate thoroughly. The mixture was centrifuged for 5 min/2500× *g* at room temperature (20–25 °C). The oil layer of the centrifuged sample was removed. A total of 1 mL of the supernatant was taken, and 9 mL of distilled water was added and mixed with the supernatant thoroughly. A total of 200 µL of this solution was taken and diluted with 9.8 mL of distilled water. Then, 100 µL of the diluted solution was applied to each well of the acylation plate. Only plastic experimental materials were used in the sample preparation. All the standards, controls, and samples were run in duplicate.

After the sample preparation, histamine was derivatized quantitatively by an acylation reagent into N-acylhistamine. The acylation procedure was carried out according to the manufacturer’s instructions using a commercial R-Biopharm Ridascreen^®^ Histamine kit (Art. No. R1601, R-Biopharm AG, Darmstadt, Germany). A total of 100 µL of each standard solution, the controls, and the sample were added to the microwells of the acylation plate. The acylation reagent and buffer were ready to use; 25 µL of acylation reagent and then 200 µL of acylation buffer were added to each microwell. The microplate was shaken by hand and incubated at room temperature (20–25 °C) for 15 min.

A total of 25 µL of the acylated standards, controls, or prepared samples was added to the microwells. After this process, 100 µL of an anti-histamine antibody solution was added to the microwells; the plate was gently mixed by shaking by hand and incubated for 40 min at room temperature (20–25 °C). The anti-histamine antibody solution used in this study was provided as part of the commercial ELISA kit (Ridascreen^®^ Histamine, Art. No. R1601, R-Biopharm AG, Darmstadt, Germany). Free acylated histamine and bound histamine competed for the antibody binding sites (competitive enzyme immunoassay). After the incubation, the liquid was removed entirely from all microwells. Then, all wells were filled with 250 µL of wash buffer, and the liquid was decanted again. The washing step was repeated twice. After washing, 100 µL of the secondary antibodies labeled with peroxidase (enzyme conjugate) was added. The plate was gently mixed by shaking by hand and incubated for 20 min at room temperature (20–25 °C). These antibodies bound to the antibody–histamine complexes. After this stage, the washing process was repeated. Any unbound enzyme-conjugated antibodies were then removed in a washing step. A total of 100 μL of substrate/chromogen was added to each microwell; the plate was gently mixed by shaking by hand and incubated in the dark for 15 min at room temperature (20–25 °C). The bound enzyme conjugate converted the chromogen into a blue product. After the incubation, 100 μL of the stop solution was added to each microwell and the plate was gently mixed by shaking by hand. The addition of the stop solution led to a color change from blue to yellow. The absorbance was measured at 450 nm against the blank within 10 min. The absorption was inversely proportional to the histamine concentration in the sample. The histamine concentration in μg/kg (ppb) corresponding to the absorbance of each sample was read from the calibration curve. The total analysis time, including the sample preparation and the incubation steps, was approximately 90 min. The test kit used in this study, as stated by the manufacturer, demonstrates an LOD of 2.5 ppm and 100% recovery and specificity for histamine in canned fish.

### 2.3. Evaluation of Daily Histamine Intake in Canned Fish

Following the analysis of histamine levels in the samples, histamine exposure due to canned fish consumption was evaluated for adult individuals. In order to estimate the health risk associated with the consumption of canned fish containing histamine, the estimated daily intake (EDI, mg/kg bw/day) was calculated using the following formula [25]:

EDI = (IR × C)/BW

where IR refers to the ingestion rate (kg/person/day), based on the assumption that an adult consumes two servings of canned fish per week [26]; C is the histamine concentration in canned fish (mg/kg); and BW represents the average body weight, which was assumed to be 70 kg for adults [27].

A risk assessment was performed by considering the maximum exposure levels of the analyzed histamines. The target hazard quotient (THQ) was calculated using the EDI values.

THQ = EDI/ARfD

where ARfD is the acute reference dose for histamine (mg/kg/day). The ARfD value for healthy individuals was determined by the European Food Safety Authority (EFSA) to be 0.714 mg/kg BW [28].

According to the risk assessment, a THQ value equal to or greater than 1 indicates a significant risk of histamine poisoning for consumers, whereas a THQ value below 1 suggests no considerable health risk.

### 2.4. Salt Analysis

The Mohr method was used to determine the amount of salt in the canned fish samples [29,30].

### 2.5. pH Analysis

A pH measurement was carried out for the canned food samples using a pH meter (Hanna, P211, Romania) directly by dipping the electrodes into the fish meat and sauce at 20 °C. The pH value was read directly from the device when a constant value was reached. After the measurements, the electrodes were made ready for reuse by wiping them with cotton soaked in diethyl ether and passing them through distilled water [31].

### 2.6. Statistical Analysis

In this study, the histamine concentrations in the canned tuna, sardine, and anchovy samples collected from different supermarkets in Ankara were measured and expressed in mg/kg. The analysis was performed using the RIDA^®^SOFT Win.NET program, which processed absorbance data obtained from the ELISA analyses and calculated the histamine levels based on standard calibration curves. Descriptive statistics, including the mean, standard error, and minimum and maximum values, were calculated for each canned fish sample. A one-way ANOVA was applied to evaluate potential differences in the pH values and salt levels of more than two groups. Independent samples *t*-tests were performed to determine the significance of the difference in histamine levels between the two sample groups in a normal distribution. All the statistical analyses were performed using SPSS version 28 and a significance level of *p* < 0.05 was considered statistically significant [32].

## 3. Results

In this study, the presence of histamine was quantitatively investigated in a total of 80 canned fish samples, including tuna (A, B, C), sardine (D, E), and anchovy (F, G) products from different firms, obtained from supermarkets in Ankara. pH and salt analyses were also performed on these samples. In this study, histamine was not detected in a total of 47 canned fish samples, including 11 tuna, 19 sardine, and 17 anchovy samples. The presence of histamine was detected in 33 (41.25%) of the 80 food samples analyzed. Among the 33 canned fish samples in which histamine was detected, the mean concentration was found to be 5.85 ± 0.67 mg/kg. The minimum and maximum values of histamine levels in the positive samples varied between 2.51 mg/kg and 20.97 mg/kg, respectively (Table 1). According to the sampling plan for histamine in canned fish specified in the Turkish Food Codex (TFC), histamine levels between 200 and 400 mg/kg are allowed in no more than 2 out of 9 samples, and none should exceed 400 mg/kg. In our study, it was seen that the histamine level of canned fish did not exceed the limit values specified in the TFC [33].

At the same time, the mean pH and salt (%) values of the 80 canned fish samples were determined as 6.09 ± 0.02 and 1.60 ± 0.05%, respectively. Detailed data on the pH levels and salt content of the samples are presented in Table 2 and Table 3, respectively.

The physical and chemical properties of canned fish are stated by the Turkish Standards Institute (TSI). In this standard, the pH of canned fish is 4.0–6.9, and the chloride rate is given as % (m/m), maximum: 2.5 [34].

According to our findings, the mean pH and salt content (%) in canned tuna, sardines, and anchovies did not exceed the limits specified by the TSI.

The histamine exposure through canned fish products, including tuna, sardine, and anchovy products, was assessed based on the EDI and THQ values (Table 4). The EDI values for all species remained relatively low, ranging between 0.002 and 0.013 mg/kg b.w./day. Correspondingly, the THQ values calculated for healthy individuals were all below the critical threshold of 1, indicating no significant health risk associated with histamine exposure through the consumption of these products.

## 4. Discussion

Sodium chloride has been reported to reduce the activity of amino acid decarboxylase, an enzyme involved in the production of biogenic amines; however, this inhibitory effect depends on both the salt concentration and the specific bacterial strains responsible for amine formation [28]. In our study, the salt content in sardines was found to be higher than in tuna. Correspondingly, the histamine level in tuna was higher than in sardines, suggesting a potential inverse relationship between the salt concentration and histamine formation.

It has been reported that, under acidic conditions (low pH), the activity of amino acid decarboxylases increases, thereby enhancing biogenic amine formation [35]. In our study, the pH value of tuna was lower than that of sardines, and correspondingly, the histamine level in tuna was found to be higher than that in sardines.

In Türkiye, various studies have focused on assessing the histamine levels in canned fish products. Gökoğlu and Varlık [36] investigated histamine in their studies and reported the total volatile basic nitrogen, trimethylamine, and pH levels in 20 canned sardine samples belonging to four different companies in Istanbul; the mean histamine concentrations ranged between 0.75 ppm and 4.46 ppm, while the mean pH values ranged from 6.28 to 6.50.

Güven and Koç [37] investigated the histamine levels in 70 frozen tuna and 160 canned tuna samples using a spectrophotometric method. They reported that the mean histamine concentration was 23 mg/kg in frozen tuna samples and 28 mg/kg in canned tuna samples. When compared with the findings of this research, the mean histamine level obtained in canned tuna in our study (7.05 ± 1.07 mg/kg) was found to be lower.

Aksu et al. [38] investigated the histamine levels in local mackerel sold in Istanbul and investigated 50 samples in total using the ELISA technique. In their study, histamine was detected at levels below 20 ppm in 32% of the mackerel samples, between 20 and 50 ppm in 42%, between 51 and 100 ppm in 18%, and between 101 and 200 ppm in three samples.

Duyar and Ekici [39] conducted histamine and pH analyses using a spectrofluorometric method on 12 canned tuna, 7 canned sardine, and 9 canned mackerel samples collected in Van, Türkiye. They reported that the mean histamine content was 21.94 ± 2.76 mg/kg with a pH of 5.68 ± 0.07 in canned tuna, 41.73 ± 0.17 mg/kg with a pH of 6.14 ± 0.17 in canned sardines, and 24.45 ± 2.38 mg/kg with a pH of 5.87 ± 0.40 in canned mackerel.

Compared to our findings, the histamine levels reported by Duyar and Ekici [39] in canned tuna and sardines were higher than the levels found in the same fish species in our study. However, the pH values of canned tuna and sardines in their study were lower than those observed in our samples.

Er et al. [40] investigated the histamine and pH values in a total of 80 canned tuna samples from four different firms (A, B, C, D) obtained from local markets in Ankara using the ELISA method. They reported that the mean histamine level in the tuna samples was 10.97 ± 9.86 mg/kg. They determined the mean pH values for firms A, B, C, and D as being 5.89 ± 0.02, 5.86 ± 0.01, 5.83 ± 0.02, and 5.82 ± 0.02, respectively. The mean histamine value reported by Er et al. [40] was found to be higher than the mean histamine value obtained in our study.

Karsandı and Bilgin [41] used the HPLC method on frozen anchovies, fresh bonito, frozen bonito rings, canned tuna, canned sardines, canned mackerel with lemon sauce, pure canned mackerel, fresh sea bass, fresh mackerel, and sauced bream fillets purchased from different markets in the Isparta province. They investigated the concentrations of biogenic amines. The researchers stated that they could not find histamine in the frozen anchovies, frozen bonito rings, or fresh mackerel and that they detected the highest histamine level in canned sardines, with a mean value of 8.16 ± 0.39 mg/100 g. The mean histamine levels reported by the researchers in the canned sardine samples were higher than the levels determined in our study.

Kızanlık et al. [42] investigated the histamine amounts using the ELISA technique in 15 bonito fish, 15 mackerel fish, and 20 horse mackerel fish samples obtained from the market in Aydın province. They reported that the mean histamine levels were 7.03 ± 1.61 ppm in bonito, 5.09 ± 1.04 ppm in mackerel, and 8.87 ± 6.61 ppm in horse mackerel.

Studies have also been conducted abroad that have investigated histamine values and some physical and chemical parameters. Gonzaga et al. [43] determined the histamine levels in 17 bonito (*Sarda sarda*), 16 mackerel (*Scomber japonicus peruanus*), and 5 mahi-mahi (*Coryphaena hippurus*) samples taken from markets in Lima, Peru, using the ELISA technique. They reported that the histamine levels were between 1 and 10 ppm in 18% of the samples (three mackerel and four bonito) and >10 ppm in 8% of the samples (three mackerel; 35 to 86 ppm).

Singh et al. [44] investigated the histamine levels in 78 fish samples (*Scomberomorus brasiliensis*, *Scomberomorus cavalla*) collected from different markets in Trinidad, West Indies, using the Max Signal histamine enzyme kit. They found the histamine levels to be ≤50 ppm in 98.7% of the fish samples.

Evangelista et al. [45] used the HPLC method to investigate the histamine levels in 135 fresh fish samples, 117 of which were fresh tuna fillets obtained from farms on the southeastern coast of Brazil and 92 of which were canned tuna samples obtained from local markets. They did not detect histamine in the fresh tuna fillets or in 41 of the 92 canned tuna samples (55.4%). However, in 41 samples (44.6%), they detected histamine at levels ranging from 0.45 to 83.73 mg/kg.

Mejrhit et al. [46] used an ELISA test to investigate the histamine content in 80 fish samples collected from various local markets in the Fez region of Morocco. They reported that the histamine levels ranged from undetectable (<1 mg/kg) to 7331 mg/kg.

In the study conducted by Simunovic et al. [23] in Serbia, the histamine levels were investigated in 819 canned tuna, 486 canned sardine, 117 canned mackerel, and 621 smoked salmon samples using the LC–MS/MS method. It was reported that histamine was detected in 22.91% of all the samples. The researchers determined the mean histamine levels in the canned tuna, canned sardine, canned mackerel, and smoked salmon samples to be 9.21, 3.16, 3.34, and 5.22 mg/kg, respectively. They also noted the highest histamine levels in canned tuna and canned mackerel, at 1112 mg/kg and 412 mg/kg, respectively. These results were higher than the mean values we obtained for canned tuna fish and lower than those we obtained for sardine fish in our study.

Weremfo et al. [5] investigated 21 canned mackerel, 14 canned sardine, and 8 canned tuna samples in Ghana for five biogenic amines (histamine, tyramine, cadaverine, putrescine, and agmatine) using the HPLC method. Tyramine was found in 42.5% of the canned fish samples, followed by putrescine (37.5%), agmatine (17.5%), histamine (15%), and cadaverine (12.5%). The researchers stated that they found the mean histamine value to be 5.65 mg/kg in canned mackerel and 5.22 mg/kg in canned tuna products, and that they did not find histamine in canned sardines. These results were lower than our study’s histamine values for canned tuna and sardine fish.

Sadeghi et al. [47] investigated the histamine content in 56 canned tuna fish samples from 22 different brands sold in Tehran markets using the ELISA method. They found that the histamine levels in the samples ranged from 2.14 ± 0.17 mg/100 g to 21.69 ± 0.11 mg/100 g. These results were higher than the histamine values we obtained for canned tuna in our study.

Harmoko et al. [48] used LC–MS/MS to investigate the histamine levels in 37 canned tuna, 16 canned sardine, and 7 canned mackerel samples obtained from Jakarta, Indonesia. The researchers reported that the samples were contaminated with histamine across a wide concentration range and exhibited high mean levels (10–53 mg/kg). Specifically, they found that the mean histamine level was 9.91 ± 0.92 mg/kg in canned tuna samples, 49.68 ± 1.34 mg/kg in canned sardine samples, and 23.12 ± 0.84 mg/kg in canned mackerel samples. The results of their study were generally higher than the histamine levels observed in our analysis for canned tuna and sardine samples.

The findings of our histamine analysis generally differed from the histamine values stated in the mentioned studies. These differences may have been due to the raw material, product type, production process, hygienic conditions, or storage conditions.

The dietary intake of histamine has been associated with histamine intolerance, a condition characterized by allergy-like symptoms, and excessive intake may result in histamine toxicity [49]. The consumption of histamine via foodstuffs in amounts of 8–40 mg, 40–100 mg, and above 100 mg has been associated with mild, moderate, and severe histamine poisoning, respectively. The reported symptoms of histamine toxicity include headaches, gastrointestinal disturbances, dermatological reactions, asthma attacks, respiratory failure, arrhythmias, and hypotension [25]. Determining the histamine levels in canned fish products and estimating the target hazard quotient based on the estimated daily histamine intake are of great importance to public health in order to assess the histamine exposure resulting from canned fish consumption by healthy and susceptible individuals.

Based on the data obtained in this study, the consumption of canned tuna, sardines, and anchovies appears to present a low risk of histamine-related health effects for the general population. Rahmani et al. [27] calculated the estimated daily intake (EDI) of histamine for adults as 0.20 mg/kg/day based on the mean histamine concentration. They reported that the mean histamine concentration in canned tuna in Iran was below the FDA standard limit, and that no histamine-related health risks were observed for adult age groups due to canned tuna consumption. According to Rachmawati et al. [50], who assessed histamine exposure and performed a semi-quantitative risk analysis of tuna and related fish species in Indonesia, the highest potential histamine exposure (based on %ARfD) was found in infants, followed by children and adults. However, they noted that the exposure levels were below the defined safety threshold, indicating a low risk of negative health effects. Petrovic et al. [51] performed a quantitative exposure assessment for fish-related histamine and heavy metals in the Serbian adult population. They reported that the mean EDI of histamine was 0.0274 mg/kg bw/day, and that 0.04% of the Serbian population was exposed to histamine through fish and seafood consumption.

## 5. Conclusions

In our study, histamine was detected in 41.25% of the 80 canned fish samples. However, the histamine amounts in the samples were within the legal limit values specified in the TFC. The pH and salt values of canned tuna, sardine, and anchovy samples were found to be within the limits specified in the TSI. Estimating the daily intake (EDI) and target hazard quotient (THQ) values based on the histamine levels in canned fish is important for determining the risks to public health, and it was found that the canned tuna, sardine, and anchovy products analyzed in our study do not pose a significant health risk due to histamine exposure in healthy individuals.

Risky foods such as canned fish samples must be monitored with effective analysis methods to prevent histamine in these foods until they reach the consumer. However, using good-quality raw materials and paying attention to hygienic production and storage conditions can prevent histamine formation in these foods. Because histamine in canned fish, which is a widely consumed ready-made food, may negatively affect human health, it is crucial to monitor histamine levels with analytical controls.

## Figures and Tables

**Table 1:** Histamine levels (mg/kg) in samples according to product group.

Product Group	N (Positive)	Mean ± SE	Minimum Levels	Maximum Levels
Tuna	30 (19)	7.05 ^a^ ± 1.07	2.62	20.97
Sardine	30 (11)	4.09 ^b^ ± 0.32	2.51	5.50
Anchovy	20 (3)	4.67 ± 0.58	3.74	5.72

^a,b^: differences between means with different letters are statistically significant (*p* < 0.05). No statistical comparison was made for anchovies due to an insufficient number of histamine-positive samples. SE: standard error.

**Table 2:** pH values of samples according to product group.

Product Group	N	Mean ± SE	Minimum Value	Maximum Value
Tuna	30	5.91 ^c^ ± 0.009	5.62	6.10
Sardine	30	6.32 ^a^ ± 0.02	5.94	6.64
Anchovy	20	5.99 ^b^ ± 0.04	5.30	6.34

^a–c^: differences between means with different letters are statistically significant (*p* < 0.05). SE: standard error.

**Table 3:** Salt (%) values of samples according to product group.

Product Group	N	Mean ± SE	Minimum Value	Maximum Value
Tuna	30	1.18 ^b^ ± 0.03	0.80	1.74
Sardine	30	1.80 ^a^ ± 0.09	1.11	4.70
Anchovy	20	1.91 ^a^ ± 0.09	0.92	3.20

^a,b^: differences between means with different letters are statistically significant (*p* < 0.05). SE: standard error.

**Table 4:** Evaluation of histamine exposure and associated risks for canned fish products.

Product Groups	EDI (mg/kg b.w./day)	THQ (Healthy Individuals)
Tuna	0.004 (0.002–0.013)	0.006 (0.002–0.018)
Sardine	0.002 (0.002–0.003)	0.003 (0.002–0.005)
Anchovy	0.003 (0.002–0.003)	0.004 (0.003–0.005)

Note: values in parentheses represent the minimum and maximum range.

## Data Availability

The original contributions presented in the study are included in the article, further inquiries can be directed to the corresponding author.
